# Is Erythrocyte Protoporphyrin a Better Single Screening Test for Iron Deficiency Compared to Hemoglobin or Mean Cell Volume in Children and Women?

**DOI:** 10.3390/nu9060557

**Published:** 2017-05-31

**Authors:** Zuguo Mei, Rafael C. Flores-Ayala, Laurence M. Grummer-Strawn, Gary M. Brittenham

**Affiliations:** 1 Division of Nutrition, Physical Activity and Obesity, National Center for Chronic Disease Prevention and Health Promotion, Centers for Disease Control and Prevention (CDC), Atlanta, 30300 GA, USA; rnf2@cdc.gov; 2 Nutrition for Health and Development, World Health Organization (WHO), Geneva 1201, Switzerland; grummerstrawnl@who.int; 3 Department of Pediatrics, Columbia University, New York, 10032 NY, USA; gmb31@cumc.columbia.edu

**Keywords:** iron deficiency, hemoglobin, erythrocyte protoporphyrin, zinc protoporphyrin, mean cell volume, serum ferritin, serum transferrin receptor, total body iron, receiver operating characteristic curve

## Abstract

Hemoglobin (Hb), mean cell volume (MCV), and erythrocyte protoporphyrin (EP) are commonly used to screen for iron deficiency (ID), but systematic evaluation of the sensitivity and specificity of these tests is limited. The objective of this study is to determine the sensitivity and specificity of Hb, MCV, and EP measurements in screening for ID in preschool children, non-pregnant women 15–49 years of age, and pregnant women. Data from the National Health and Nutrition Examination Surveys (NHANES) (NHANES 2003–2006: *n* = 861, children three to five years of age; *n* = 3112, non-pregnant women 15 to 49 years of age. NHANES 1999–2006: *n* = 1150, pregnant women) were examined for this purpose. Children or women with blood lead ≥10 µg/dL or C-reactive protein (CRP) >5.0 mg/L were excluded. ID was defined as total body iron stores <0 mg/kg body weight, calculated from the ratio of soluble transferrin receptor (sTfR) to serum ferritin (SF). The receiver operating characteristic (ROC) curve was used to characterize the sensitivity and specificity of Hb, MCV, and EP measurements in screening for ID. In detecting ID in children three to five years of age, EP (Area under the Curve (AUC) 0.80) was superior to Hb (AUC 0.62) (*p* < 0.01) but not statistically different from MCV (AUC 0.73). In women, EP and Hb were comparable (non-pregnant AUC 0.86 and 0.84, respectively; pregnant 0.77 and 0.74, respectively), and both were better than MCV (non-pregnant AUC 0.80; pregnant 0.70) (*p* < 0.01). We concluded that the sensitivity and specificity of EP in screening for ID were consistently superior to or at least as effective as those of Hb and MCV in each population examined. For children three to five years of age, EP screening for ID was significantly better than Hb and similar to MCV. For both non-pregnant and pregnant women, the performance of EP and Hb were comparable; both were significantly superior to MCV.

## 1. Introduction

Worldwide, according to the World Health Organization (WHO) [1], iron deficiency (ID) affects more individuals than any other health problem, with children and women of childbearing age at the greatest risk [2,3]. ID can be detected by hematologic tests, including hemoglobin (Hb), hematocrit, mean cell volume (MCV), red cell distribution width, and reticulocyte hemoglobin content, or by biochemical tests, including serum iron (Fe), total iron binding capacity, transferrin saturation (TS), serum ferritin (SF), serum transferrin receptor (sTfR), and erythrocyte protoporphyrin (EP). Among these tests, Hb is most commonly used to screen for anemia as a proxy for ID because of its low cost, better performance than hematocrit, and the availability of easy and rapid procedures for measurement [4,5,6]. Hb is a late indicator, detecting ID only after the lack of iron has reduced the circulating hemoglobin concentration below thresholds used for anemia [7,8,9]. In addition, Hb lacks specificity because of the variety of other conditions that can be responsible for anemia. Biochemical tests are more sensitive and specific for ID but generally are more costly and complicated to perform than hematologic tests. Of all the biochemical tests, EP may have the most potential as an inexpensive and simple screening test because erythrocyte zinc protoporphyrin (ZnPP), the protoporphyrin that is predominantly increased with ID, can be measured with a portable hematofluorometer [7] and a non-invasive method is under development [10].

In the US, several methods have been used to define ID. The most common of these were the models that classified individuals as ID when abnormal values were present in at least two of three measures of either SF, TS, and EP (the ferritin model) or MCV, TS, and EP (the MCV model) [8,9,11]. The justification for the use of a combination of tests was the finding that populations with only one abnormal test of these three had scarcely more anemia than those with all test results within reference ranges [9,12]. In 2003, Cook et al. [13,14] introduced a method for the assessment of total body iron (TBI) that is based on the ratio of sTfR to SF. This quantitative estimate, which expresses TBI stores on the basis of body weight, is suggested to allow for an evaluation of the full range of iron status from deficiency to excess within a population. The suggested TBI cutoff value for defining ID is <0 mg/kg [13,14]. This quantitative approach provides information on the full range of body iron stores beyond that available from the ferritin model or MCV model, which were traditionally used to assess the prevalence if ID in the US population [15,16]. TBI has been adopted for the interpretation of National Health and Nutrition Examination Survey (NHANES) data [15,16] and for monitoring ID in the US population for US Healthy People 2020 [17].

In our earlier analysis [18] of data from the third NHANES (1988–1994), we used two of three abnormal values of ferritin, TS, and MCV to define ID and compared the sensitivity and specificity of EP and Hb for detecting ID using receiver operating characteristic (ROC) curves. We found that EP was a better screening tool for ID than Hb among children aged one to five but had similar sensitivity and specificity for predicting ID among non-pregnant women aged 15–49 [18]. Here, we update the analysis of these data using the definition of ID by quantitative estimation of TBI stores calculated from the ratio of sTfR to SF that has now been adopted by NHANES [15,16]. We compare the performance of EP, Hb, and MCV in detecting ID in children aged three to five years, non-pregnant women aged 15–49 years, and pregnant women.

## 2. Materials and Methods

NHANES is a multi-purpose survey designed to assess the health and nutritional status of adults and children in the United States. Currently NHANES is a continuous survey (1999–present) that includes an interview in the household followed by a standardized physical examination in a Mobile Examination Center. The NHANES relies on a stratified multistage probability sample that is based on the selection of counties, blocks, households, and persons within households. The surveys were conducted by the National Center for Health Statistics (NCHS), Centers for Disease Control and Prevention (CDC). Ethical approval was obtained by the NCHS Research Ethics Review Board. Written informed consent was obtained from participants 12 years and older. Parental consent was obtained for those under age 18. The procedures for data collection and analysis are published elsewhere [19,20,21,22]. In NHANES 2003 and later, EP was only measured in children aged three to five years and women 15–49 years of age. For the purpose of the present analysis, we combined NHANES 2003–2006 data for children three to five years old and non-pregnant women aged 15–49. To obtain an adequate sample size for pregnant women, we pooled data from 1999 to 2006.

We restricted our study sample to participants who attended physical examinations in Mobile Examination Centers; 1241 children aged three to five years, 3451 non-pregnant women aged 15–49 years, and 1219 pregnant women. We excluded those who had (i) missing SF, sTfR, EP, Hb, MCV, blood lead, or C-reactive protein (CRP) measurements (*n* = 379 for children, *n* = 334 for non-pregnant women, and *n* = 70 for pregnant women); (ii) elevated blood lead levels ≥10 µg/dL (*n* = 9 for children, *n* = 4 for non-pregnant women, and *n* = 0 for pregnant women); or (iii) elevated CRP >5.0 mg/L (*n* = 0 for children, *n* = 9 for non-pregnant women, and *n* = 8 for pregnant women). Our final sample included 853 children, 3104 non-pregnant women, and 1141 pregnant women. Sincewe did not intend to perform population-based studies, the original sample weights assigned to the data were not included in the analysis. 

sTfR and SF assays were conducted at the National Center of Environmental Health (NCEH), CDC for the 2003–2006 specimens and the surplus specimens from 1999 to 2002. In brief, the Tina-quant^®^ sTfR assay (Roche Diagnostics, Mannheim, Germany), an automated homogeneous immunoturbidimetric assay, was performed on a Hitachi 912 clinical analyzer (Roche Diagnostics, Indianapolis, IN, USA) [23]. The methodological details were described earlier [13].

SF was measured using two methods. A single-incubation two-site immunoradiometric assay (IRMA) (BioRad Laboratories, Hercules, CA, USA) was used in 2003. However, this assay was discontinued by the manufacturer in early 2004, so ferritin was measured by the Roche Tina-quant^®^ Ferritin immunoturbidimetric assay on the Hitachi 912 clinical analyzer (Roche Diagnostics, Indianapolis, IN, USA) [21] in 2004–2006. The same Roche method as used in 2004–2006 was used to analyze the surplus specimens from pregnant women in NHANES 1999–2002 [19,20]. Due to method differences between the BioRad and Roche ferritin assays, it is necessary for the concentrations obtained for 2003 samples using the BioRad assay to be statistically adjusted to be fully comparable to those obtained for the 2004 samples using the Roche assay for NHANES 2003–2004. This was accomplished prior to the data release by NCHS by applying three piecewise linear regression equations, described in detail elsewhere [21].

EP was measured at CDC NCEH before 2001 and subsequently at the State of New York Department of Health after 2001. A modification of the acid extraction method originally described by Sassa et al. [24] and Chisolm and Brown [25] was used. Protoporphyrin is first extracted from ethylenediaminetetraacetic acid (EDTA)-whole blood into a 2:1 (*v/v*) mixture of ethyl acetate-acetic acid and finally back-extracted into diluted hydrochloric acid. The protoporphyrin in the aqueous phase is measured fluorometrically at excitation and emission wavelengths of 404 and 658 nm, respectively. Calculations are based on a processed protoporphyrin IX (free acid) standard curve. After a correction for the individual hematocrit is made, the final concentration of protoporphyrin in a specimen was expressed as micrograms per deciliter of packed red blood cells (μg/dL RBC) [16,17,18,19]. For our study, we converted the unit of measurement to the preferred unit of µmol EP/mol heme by multiplying by 50 [26].

Hb and MCV were measured as part of a complete blood count in the Mobile Examination Centers using the Beckman Coulter MAXM hematology flow cytometer (Beckman Coulter, Inc., Fullerton, CA, USA) [19,20,21,22]. Whole blood lead concentration was determined using inductively coupled plasma mass spectrometry. This multi-element analytical technique is based on quadrupole inductively coupled plasma mass spectrometry (ICP-MS) technology [19,20,21,22].

A trimester was based on the number of months pregnant reported by the mother from the surveys. Only females who reported that they were pregnant at the time of the medical examination were asked about their trimester. First, second, and third trimester were defined as less than or equal to three months pregnant, four to six months pregnant, and seven months or more pregnant, respectively. The trimester value for women who did not know, were not asked, or did not report how long they had been pregnant was categorized as unknown.

Race/ethnicity was based on self-reported data from the surveys. We excluded comparisons of participants who reported that they were of Latino ethnicity other than Mexican-American and participants who reported they were from more than one race/ethnic group because of the small sample size of these groups. 

We defined ID as TBI stores <0 mg/kg [13,14]. Positive values of total body iron stores indicate the presence of surplus iron in stores and negative values indicate the extent of the iron deficit in tissues. TBI stores were calculated as described previously in detail [15,16] from sTfR and SF concentrations using the formula from Cook et al. [13,14] after converting Roche sTfR concentrations to those equivalent to the Flowers assay [27] used in the development of the total body iron stores model [13,14].

TBI stores (mg/kg) = − [log_10_ (sTfR × 1000 ÷ SF) – 2.8229] ÷ 0.1207

To convert the Roche sSTfR concentrations to those equivalent to the Flowers assay [27], we applied a conversion equation derived from a previous comparison [28] of the two assays (*n* = 40): Flowers sSTfR = 1.5 × Roche sSTfR + 0.35 mg/L. We used the original Roche SF concentrations for the total body iron stores calculation because a previous comparison of the Roche assay with the ELISA method used to develop the total body iron stores model [13,14] indicated that these two methods generated similar values [15,16].

We used ROC curves [29] to characterize the sensitivity and specificity of EP, Hb, and MCV measurements in screening for ID. The ROC curve is constructed by first calculating the sensitivity and specificity generated by using a series of EP, Hb, or MCV thresholds against the definition for ID. Then, the series of sensitivities were plotted on the y-axis against the corresponding values of 100-specificity on the x-axis. In general, the farther the curve is away from the diagonal chance line, which extends at 45° from the origin (*x* = 0, *y* = 0), the better the performance of the indicator. Statistical Analysis Software (SAS) software (version 9.3, SAS Institute Inc., Cary, NC, USA) with logistic procedures was used to generate the ROC curves and to test the significance for the areas under the ROC curves (AUC).

First, we examined the ROC performances on overall subjects for children three to five years of age, non-pregnant women 15–49 years of age, and pregnant women. As described above, we excluded subjects with elevated blood lead (>10 μg/dL) or CRP >5 mg/L because elevated EP could be caused by lead poisoning and SF is an acute-phase protein that could be elevated by inflammation. Second, we stratified our analysis by race/ethnicity for children, non-pregnant women, and pregnant women; by gender for children; and by trimester for pregnant women. Finally, because liver disease, infection, and inflammation can influence biochemical indices [30], we repeated the above analysis on a subsample that excluded subjects who had signs of infection, as indicated by abnormal white blood cell counts (>10.0 × 10^9^/L), or of possible liver disease, as defined by at least one of two abnormal elevations (more than two times the upper limit of normal value) on alanine aminotransferase (>70 U/L) and aspartate aminotransferase (>70 U/L) [31]. 

## 3. Results

The demographic and biochemical characteristics of the selected sample of the NHANES data are shown separately for children (three to fiev years of age), non-pregnant women (15–49 years of age), and pregnant women in Table 1. The prevalence of ID, defined as TBI stores ≤0 mg/kg body weight, was lowest among the preschool aged children (3.8%), intermediate among non-pregnant women (12.1%), and highest among pregnant women (21.5%).

**Figure 1:**
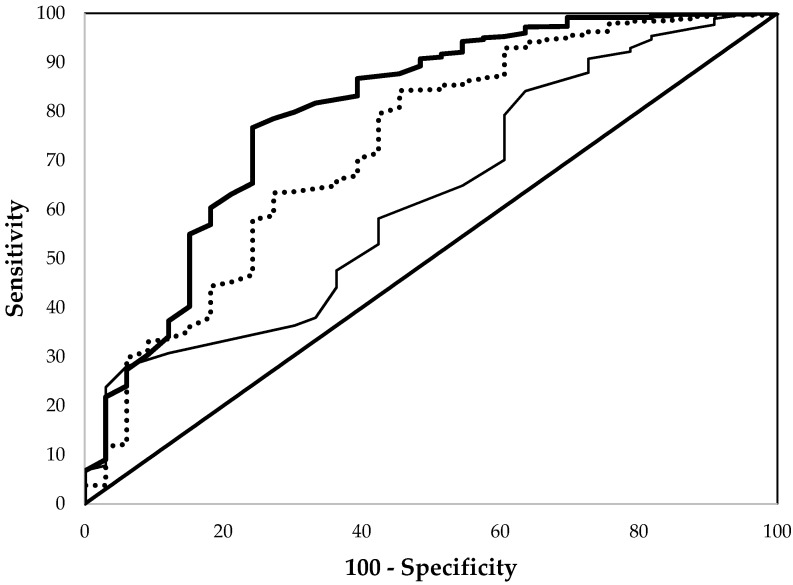
Comparison of the receiver operating characteristic curves of erythrocyte protoporphyrin (thick line), hemoglobin (thin line), and mean cell volume (dashed line) in detecting iron deficiency for children aged three to five years from the National Health and Nutrition Examination Surveys 2003–2006.

Among all preschool aged children (three to five years), the overall sensitivity and specificity of EP in detecting ID were significantly superior to Hb (*p* < 0.001) but similar to those of MCV (Figure 1, Table 2). For example, at thresholds giving 80% specificity (100-specificity of 20%), the sensitivities were about 63% for EP and 45% for MCV but only 36% for Hb for the detection of ID (Figure 1). As listed in Table 2, among male, non-Hispanic white, and Mexican American children, the results were similar. For female children, the ROC performance of EP was significantly better than those of Hb and MCV. For non-Hispanic black children, no significant differences were found among the three indices.

**Figure 2:**
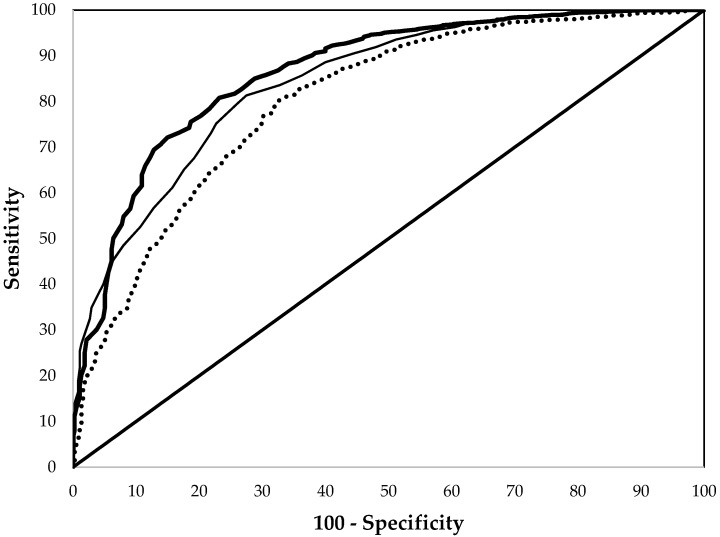
Comparison of the receiver operating characteristic curves of erythrocyte protoporphyrin (thick line), hemoglobin (thin line), and mean cell volume (dashed line) in detecting iron deficiency for non–pregnant women aged 15–49 years from the National Health and Nutrition Examination Surveys 2003–2006.

Among all non-pregnant women, the ROC performance of EP and Hb did not differ significantly, while that of MCV was significantly worse (Figure 2 and Table 2). For example, at cut points giving 80% specificity, the sensitivities were 77% for EP and 67% for Hb but only 61% for MCV for the detection of ID. Similar results were found in non-Hispanic black women, but the three indices performed similarly in non-Hispanic white and Mexican American women (Table 2).

**Figure 3:**
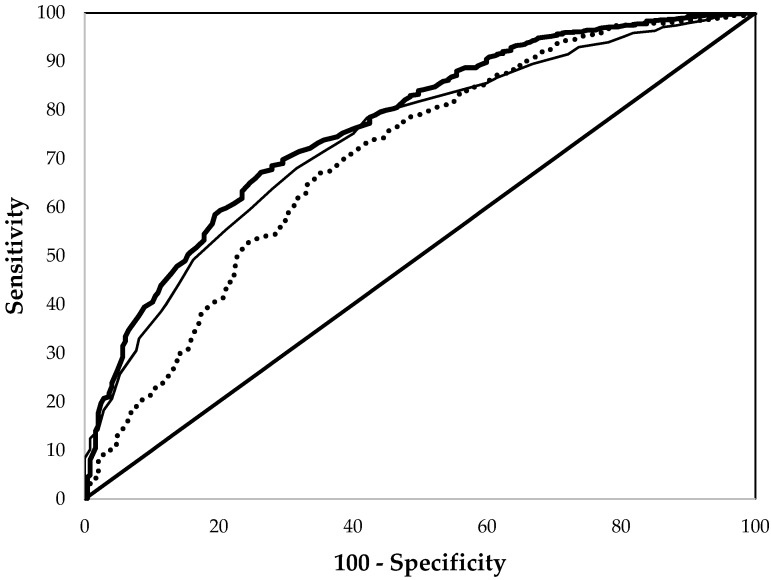
Comparison of the receiver operating characteristic curves of erythrocyte protoporphyrin (thick line), hemoglobin (thin line), and mean cell volume (dashed line) in detect iron deficiency for pregnant women aged 15–49 years from the National Health and Nutrition Examination Surveys 1999–2006.

For all pregnant women, the the AUCs in the ROC analyses were consistently lower than those in non-pregnant women (Table 2). Still, as for non-pregnant women, the ROC performance of EP and Hb did not differ significantly, while that of MCV was significantly worse (Figure 3 and Table 2). Considering sub-groups, among non-Hispanic black pregnant women the ROC performance of EP was significantly better than that of Hb and MCV, but no significant differences were found between the three measures in non-Hispanic white or Mexican American pregnant women. Considering results by trimester, the ROC performance of EP and Hb were better than that of MCV only during the first trimester; during the remainder of pregnancy the AUCs of the three measures did not differ significantly.

Finally, we repeated these analyses on subsamples of the children and pregnant and non-pregnant women after excluding subjects who had signs of infection or possible liver disease. The overall results were similar to those described above (Table 3).

## 4. Discussion and Conclusions

These results show that the sensitivity and specificity of EP in screening for ID, defined as TBI stores ≤0 mg/kg body weight, were consistently superior to or at least as effective as those of Hb and MCV, both in each population overall and within each subgroup examined. In contrast, the usefulness of Hb and MCV depended upon the population studied. Compared to EP, the screening effectiveness of Hb was worse in children but not significantly different in women. Conversely, the screening effectiveness of MCV was inferior to EP in women but not significantly different in children. The biological bases for these differences in screening performance are not evident but are likely the result of the physiological differences among these indicators. EP, as detailed more fully below, reflects the balance between erythropoietic and tissue iron requirements and the iron supply from stores and absorption. EP is unaffected by simple iron depletion and begins to increase only with the onset of iron-deficient erythropoiesis, as the iron supply becomes inadequate for red blood cell production [32]. EP then rises continuously in concert with the severity of the lack of iron. Increases in the amount of EP within newly produced erythrocytes alter the mean EP concentrations in the peripheral blood only slowly as young red blood cells replace those at the end of their life span at a rate of roughly 1% per day. EP increases before Hb and MCV begin to decline and is the first measurable biochemical change in erythrocytes with the onset of iron-deficient erythropoiesis [32]. With further decreases in TBI stores, Hb and MCV decline in a similar fashion, as newly formed erythrocytes supplant senescent cells. In children three to five years of age, the most common cause of ID is the result of the increased iron requirements of growth exceeding the amounts of bioavailable dietary iron. Between the ages of three and five years, the increase in the reference range for Hb is greater than that for MCV [33], and this greater variability may contribute to the decreased ROC performance of Hb in screening for ID. In adult women, ID is predominantly the result of iron losses with menstruation and pregnancy in excess of the amounts of bioavailable iron in the diet. Possibly the intermittent monthly losses of blood result in greater variability in MCV in the red blood cells produced immediately after menses than in Hb, the variability of which may be determined over a longer term. Conceivably, these differences could account for the lesser sensitivity and specificity of MCV in women. Whatever the underlying physiological mechanisms, our findings may help guide the choice of screening tests in planning surveys or studies in other populations in which ID is the predominant cause of anemia.

The strengths of our comparative study include: (i) the use of a broad sample of U.S. preschool children and women of childbearing age from the NHANES studies; (ii) hematologic and biochemical measurements using carefully controlled and validated assays; (iii) systematic evaluation of the screening performance of the indicators by ROC analysis; and (iv) defining ID as TBI stores <0 mg/kg as calculated from the ratio of sTfR to SF, as used for monitoring ID in the US population [17]. Previous comparisons have found fair to good agreement between estimates of the prevalence of ID using this quantitative estimate of TBI stores [15,16] and the ferritin model that we used in an earlier comparison of the screening performance of EP and Hb in children and non-pregnant women [18]. Consequently, the concordance between defining ID by calculated TBI stores and by the ferritin model may underlie the similar findings of the two approaches, i.e. that EP was superior to Hb in screening for ID in preschool children but had a comparable sensitivity and specificity in non-pregnant women [18].

Despite the many advantages of the use of the ratio of sTfR to SF to define ID [13,15,16], some remaining uncertainties must be acknowledged. First, we cannot compare our findings with an established standard such as bone marrow examination because no reference method is available for the diagnosis of ID at the population level [15]. Second, the relationship between the ratio of sTfR to SF and TBI stores was established in a single study of repeated phlebotomy in 14 healthy white men and women over six to 22 weeks [14]. The extent to which the changes in sTfR and SF during the relatively rapid reduction in body iron stores with phlebotomy resemble the more gradual diminution with nutritional ID has not been determined. Furthermore, for both ethical and practical reasons, neither children nor pregnant women were included in the single study so the only evidence of the validity of this means of estimating TBI stores in these groups is indirect [15]. Third, sTfR and SF respond more rapidly to changes in body iron deficits than do EP, Hb, and MCV, which change only slowly as senescent erythrocytes are replaced by red blood cells that are the product of iron-deficient erythropoiesis. Accordingly, the ratio of sTfR to SF can reflect ID before the mean EP, Hb, and MCV in the peripheral blood are outside their reference ranges. Fourth, we aimed to limit the effect of inflammation or liver disease on raising SF independently of an increase in TBI stores by excluding subjects with elevated blood lead, CRP, white blood cell count, or liver transferases. This effort may have been incompletely effective, resulting in the misclassification of some iron-deficient subjects as iron replete. Due to their higher prevalence of recent acute infection, the results in children three to five years of age were most likely to have been affected by such misclassification. For example, in a study of red blood cell protoporphyrin and serum ferritin in U.S. preschool children, more than half the children had evidence of a recent illness, but CRP was elevated in only 12.4% [34].

EP was measured in the NHANES studies using a modification of an acidic extraction method [24]. Until the early 1970s, the protoporphyrin extracted with such methods was referred to as “free erythrocyte protoporphyrin” or simply “erythrocyte protoporphyrin”, and was considered to be the form of protoprophyrin within erythrocytes. In 1974, the predominant form of protoporphyrin in normal red blood cells and in red blood cells from patients with ID was conclusively shown to be zinc protoporphyrin [35]. The extraction methods using acidic solvents had released the zinc from zinc protoporphyrin, unknowingly producing protoporphyrin IX [35,36]. In the developing red blood cell, the incorporation of iron into protoporphyrin IX is the terminal step in the formation of heme for the synthesis of hemoglobin. If iron is unavailable, divalent zinc is inserted instead, producing zinc protoporphyrin, which persists for the life of the erythrocyte. In normal red blood cells, zinc protoporphyin constitutes up to 90% or more of the non-heme protoporphyrin, with the remainder present as protoporphyrin IX [26]. With ID and inflammation, the increased protoporphyrin is predominantly zinc protoporphyrin [32]. As is indicated in these studies [26,32], because approximately 90% or more of the erythrocyte protoporphyrin measured in the NHANES studies was in fact zinc protoporphyrin, the acid extraction method still provided an effective means to screen for ID. At present, zinc protoporphyrin can be measured directly in a drop of blood using a portable hematofluometer [7], and an optical method not requiring a blood sample has been described [10]. Conceivably, the direct measurement of zinc protoporphyrin might be more sensitive and specific in screening for ID than the determination of EP by acidic extraction, but this possibility has not been examined directly.

The results of our comparative analysis of EP, Hb, and MCV seem robust for the U.S., where ID is the most common single cause of anemia among young children and women of childbearing age [37,38]. Caution should be used in generalizing the results to other settings in which these indicators, as well as the ratio of sTfR to SF, may be affected by other conditions independently of TBI stores. Each may be altered by malaria and other infections, inflammatory disorders, or by some combination of these conditions. In particular, EP may be increased by exposure to lead or other heavy metals [32,39] and by certain rare or uncommon genetic and acquired disorders, including mutations in the gene KLF1 (Kruppel-Like Factor 1 (Erythroid)) [40], sideroblastic and inherited microcytic anemias [41], myelodysplasia [42] and some forms of porphyria [43]. With sickle-cell traits, EP levels are normal but may be raised in sickle cell anemia in the subset of those with fetal hemoglobin levels <9% [44]. The effect of hemoglobinopathies has not been well characterized; no elevations with alpha- or beta-thalassemia trait were described in some studies [45,46,47], while modest elevations, overlapping with levels found with mild ID, have been reported in iron-replete subjects with alpha- or beta-thalassemia traits and with hemoglobin E [48,49,50,51]. Hemoglobinopathies will also alter the use of MCV in screening for ID. In populations in which anemia may be the result of conditions that are uncommon in the U.S., such as other nutritional deficiencies (e.g., vitamin A, vitamin B_12_ or folate deficiency), malaria, and other infections, Hb may be so non-specific as to be of little use in screening for ID [4,52]. 

In conclusion, using the criterion for ID of TBI stores <0 mg/kg body weight, as estimated from the ratio of sTfR to SF, the sensitivity and specificity of EP in screening for ID were consistently superior to or at least as effective as those of Hb and MCV in each population examined. For children three to five years of age, EP screening for ID was significantly better than Hb screening and similar to MCV screening. For both non-pregnant and pregnant women, the performance of EP and Hb were comparable; both were significantly superior to MCV. As in our earlier study [18], we conclude that the measurement of red blood cell zinc protoporphyrin, which should correlate closely with EP, by using portable hematofluorometers deserves further consideration as a field assay for screening for ID.

## Figures and Tables

**Table 1:** Basic demographic and biochemical characteristics of the selected sample of children (three to five years old), non-pregnant women (15–49 years), and pregnant women from the National Health and Nutrition Examination Surveys (NHANES, 2003–2006 for children and non-pregnant women, 1999–2006 for pregnant women) ^1^.

	Children (*n* = 853)	Non-Pregnant Women (*n* = 3104)	Pregnant Women (*n* = 1141)
% of female	48.8	100	100
Age (years) ^2^	4.0 ± 0.8	28.4 ± 11.1	26.3 ± 5.7
% of Non-Hispanic white	27.6	39.0	44.0
% of Non-Hispanic black	31.0	28.3	15.6
% of Mexican-American	29.1	24.4	29.7
Others	12.3	8.3	10.7
EP (μmol/L) ^2^	0.91 ± 0.36	1.13 ± 0.72	1.26 ± 0.62
Hb (g/dL) ^2^	12.77 ± 0.80	13.40 ± 1.17	12.36 ± 1.04
MCV (fL) ^2^	82.05 ± 4.34	88.09 ± 5.98	89.40 ± 5.42
SF (μg/L) ^2^	29.70 ± 1.72	34.26 ± 2.51	19.58 ± 2.54
sTfR (mg/L) ^2^	4.21 ± 1.24	3.62 ± 1.41	3.35 ± 1.42
TBI (mg/kg) ^2^	3.91 ± 2.22	4.92 ± 4.04	3.17 ± 4.10
Lead (μg/dL)	2.01 ± 1.41	1.10 ± 0.86	0.94 ± 0.75
CRP (mg/L) ^2^	0.04 ± 4.06	0.15 ± 4.42	0.48 ± 2.74
% anemia ^3^	1.4	9.1	5.6
% TBI < 0 mg/kg	3.9	12.1	21.6

^1^ EP, erythrocyte protoporphyrin; Hb, hemoglobin; MCV, mean cell volume; SF, serum ferritin; sTfR, soluble transferrin receptor; TBI, total body iron; CRP, C-reactive protein. ^2^ Mean ± SD. Arithmetic means for EP, Hb, MCV, TBI, and Lead; Geometric means for SF, sTfR, and CRP. ^3^ On the basis of the Centers for Disease Control and Prevention hemoglobin cutoffs [6].

**Table 2:** Comparison of the areas under the ROC curves (Mean ± SE) of erythrocyte protoporphyrin (EP), hemoglobin (Hb), and mean cell volume (MCV) in detecting iron deficiency in children (three to five years old), non-pregnant women (15–49 years), and pregnant women from the National Health and Nutrition Examination Surveys (NHANES, 2003–2006 for children and non-pregnant women, 1999–2006 for pregnant women) ^1^.

	Children (*n* = 853) ^2^	Non-Pregnant Women (*n* = 3104) ^2^	Pregnant Women (*n* = 1141) ^2^
**Total sample**			
EP	0.799 ± 0.046 ^a^	0.864 ± 0.010 ^a^	0.766 ± 0.016 ^a^
Hb	0.624 ± 0.050 ^b^	0.843 ± 0.011 ^a^	0.740 ± 0.017 ^a^
MCV	0.728 ± 0.050 ^a^	0.802 ± 0.013 ^b^	0.700 ± 0.020 ^b^
**Male**			
EP	0.787 ± 0.066 ^c^	-	-
Hb	0.635 ± 0.067 ^d^	-	-
MCV	0.768 ± 0.067 ^c^	-	-
**Female**			
EP	0.825 ± 0.054 ^a^	0.864 ± 0.010 ^a^	0.766 ± 0.016 ^a^
Hb	0.607 ± 0.076 ^b^	0.843 ± 0.011 ^a^	0.740 ± 0.017 ^a^
MCV	0.669 ± 0.067 ^b^	0.802 ± 0.013 ^b^	0.700 ± 0.020 ^b^
**Non-Hispanic white**			
EP	0.806 ± 0.069 ^c^	0.878 ± 0.019 ^a^	0.778 ± 0.028 ^a^
Hb	0.599 ± 0.115 ^d^	0.878 ± 0.018 ^a^	0.783 ± 0.027 ^a^
MCV	0.730 ± 0.090 ^c^	0.836 ± 0.022 ^a^	0.726 ± 0.033 ^a^
**Non-Hispanic black**			
EP	0.665 ± 0.127 ^a^	0.882 ± 0.018 ^a^	0.828 ± 0.034 ^a^
Hb	0.495 ± 0.106 ^a^	0.843 ± 0.019 ^a^	0.667 ± 0.042 ^b^
MCV	0.575 ± 0.117 ^a^	0.761 ± 0.025 ^b^	0.653 ± 0.049 ^b^
**Mexican American**			
EP	0.821 ± 0.066 ^c^	0.829 ± 0.020 ^a^	0.744 ± 0.030 ^a^
Hb	0.677 ± 0.071 ^d^	0.830 ± 0.021 ^a^	0.753 ± 0.029 ^a^
MCV	0.781 ± 0.082 ^c^	0.815 ± 0.022 ^a^	0.688 ± 0.034 ^a^
**1st trimester**			
EP	-	-	0.873 ± 0.053 ^c^
Hb	-	-	0.764 ± 0.066 ^c^
MCV	-	-	0.730 ± 0.080 ^d^
**2nd trimester**			
EP	-	-	0.744 ± 0.032 ^a^
Hb	-	-	0.746 ± 0.031 ^a^
MCV	-	-	0.720 ± 0.036 ^a^
**3rd trimester**			
EP	-	-	0.715 ± 0.028 ^a^
Hb	-	-	0.722 ± 0.027 ^a^
MCV	-	-	0.742 ± 0.027 ^a^

^1^ EP, erythrocyte protoporphyrin; Hb, hemoglobin; MCV, mean cell volume. To generate the ROC curves, EP was converted to the preferred unit of µmol EP/mol heme by multiplying by 50. ^2^ Within a group, values with different superscript letters (a or b) are significantly different (*p* < 0.01) in the areas under the curves, and values with different superscript letters (c or d) are significantly different (*p* < 0.05) in the areas under the curves.

**Table 3:** Comparison of the areas under the receiver operating characteristic (ROC) curves (Mean ± SE) of erythrocyte protoporphyrin (EP), hemoglobin (Hb), and mean cell volume (MCV) in detecting iron deficiency in children (three to five years p;d), non-pregnant women (15–49 years), and pregnant women from the National Health and Nutrition Examination Surveys (NHANES, 2003–2006 for children and non-pregnant women, 1999–2006 for pregnant women), after excluding subjects who had signs of infection, as indicated by abnormal white blood cell counts (>10.0 × 10^9^/L) or elevated C-reactive protein (>5 mg/L) or of possible liver disease, as defined by at least one of two abnormal elevations (more than two times the upper limit of normal value) on alanine aminotransferase (>70 U/L) and aspartate aminotransferase (>70 U/L) ^1^.

	Children (*n* = 762) ^2^	Non-Pregnant Women (*n* = 2746) ^2^	Pregnant Women (*n* = 684) ^2^
EP	0.785 ± 0.049 ^a^	0.864 ± 0.011 ^a^	0.771 ± 0.022 ^a^
Hb	0.596 ± 0.054 ^b^	0.841 ± 0.011 ^a^	0.743 ± 0.022 ^a^
MCV	0.703 ± 0.052 ^a^	0.802 ± 0.014 ^b^	0.698 ± 0.026 ^b^

^1^ EP, erythrocyte protoporphyrin; Hb, hemoglobin; MCV, mean cell volume. To generate the ROC curves, EP was converted to the preferred unit of µmol EP/mol heme by multiplying by 50. ^2^ Values with different superscript letters (a or b) are significantly different (*p* < 0.01) in the areas under the curves.

## Abbreviations

ID	iron deficiency
Hb	hemoglobin
EP	erythrocyte protoporphyrin
MCV	mean cell volume
TS	transferrin saturation
SF	serum ferritin
sTfR	serum transferrin receptor
ZnPP	zinc protoporphyrin
TBI	total body iron
CRP	C-reactive protein
NHANES	National Health and Nutrition Examination Surveys
ROC	receiver operating characteristic
